# Segmentation, tracking, and sub-cellular feature extraction in 3D time-lapse images

**DOI:** 10.1038/s41598-023-29149-z

**Published:** 2023-03-01

**Authors:** Jiaxiang Jiang, Amil Khan, S. Shailja, Samuel A. Belteton, Michael Goebel, Daniel B. Szymanski, B. S. Manjunath

**Affiliations:** 1 Department of Electrical and Computer Engineering, University of California, Santa Barbara, USA; 2 Department of Botany and Plant Pathology, Purdue University, West Lafayette, USA; 3 Molecular Biology Program, New Mexico State University, Las Cruces, USA

**Keywords:** Plant cell biology, Software, Computational science

## Abstract

This paper presents a method for time-lapse 3D cell analysis. Specifically, we consider the problem of accurately localizing and quantitatively analyzing sub-cellular features, and for tracking individual cells from time-lapse 3D confocal cell image stacks. The heterogeneity of cells and the volume of multi-dimensional images presents a major challenge for fully automated analysis of morphogenesis and development of cells. This paper is motivated by the pavement cell growth process, and building a quantitative morphogenesis model. We propose a deep feature based segmentation method to accurately detect and label each cell region. An adjacency graph based method is used to extract sub-cellular features of the segmented cells. Finally, the robust graph based tracking algorithm using multiple cell features is proposed for associating cells at different time instances. We also demonstrate the generality of our tracking method on *C. elegans* fluorescent nuclei imagery. Extensive experiment results are provided and demonstrate the robustness of the proposed method. The code is available on GitHub and the method is available as a service through the BisQue portal.

## Introduction

The sizes and shapes of leaves are key determinants of the efficiency of light capture in plants, and the overall photosynthetic rates of the canopy is a key determinant of yields^1^. The rates and patterns of leaf expansion are governed by the epidermal tissue^2^ but understanding how the irreversible growth properties of its constituent jig-saw-puzzle piece cells related to organ level shape change remains as a major challenge.

**Figure 1:**
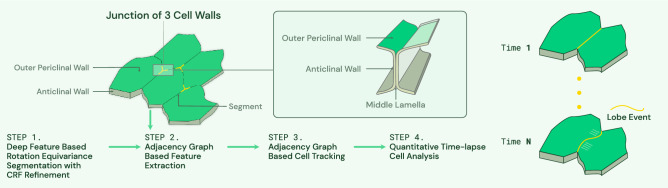
Workflow of proposed method. Modified from^4^. Given a sequence of 3D image stacks, deep feature based rotation equivariance deep learning model with CRF refinement is used to segment each cell. Then adjacency graph is built based on segmented image and used for sub-cellular feature extraction and tracking. Sub-cellular features such as junction of three cell walls and anticlinal wall segment are illustrated in the figure. Next detected segments will be used in^5^ to detect lobes. This paper mainly focuses on Step 1 to Step 3.

The epidermal cell, also known as pavement cell, undergoes a dramatic transformation from a slightly irregular polyhedral cell to a highly convoluted and multi-lobed morphology. The interdigitated growth mode is widespread in the plant kingdom^3^, and the process by which lobing occurs can reveal how force patterns in the tissue are converted into predictable shape change^4^. To analyze the slow and irreversible growth behavior across wide spatial scales, it is important to track and map lobing events in the epidermal tissue. It has been shown that cell walls perpendicular to the leaf surface, the anticlinal wall as illustrated in Fig. 1, can be used to detect new lobe formations^5,6^.

Time-lapse image stacks from 3D confocal imagery provide a good resource to study the pavement cell growth process, and build the quantitative cell morphogenesis model^4,7^. 3D confocal microscopy data contain large amount of cell shape and sub-cellular cell wall structure information. Cell analysis requirements include detecting sub-cellular features such as junctions of three cell walls and segments shape of anticlinal cell walls used to detect lobes, all of which depends on accurate segmentation. These sub-cellular features are illustrated in Fig. 1. Currently, these features are usually acquired manually from 3D image stacks. Manual extraction and analysis is not only laborious but also prevents evaluation of large amounts of data necessary to map relationships between lobe formation to leaf growth.

Existing automatic time-lapse cell analysis methods include mainly two steps: (1) Recognizing and localizing cells and cell walls spatially (segmentation) and tracking cells in temporal dimension, (2) cellular/sub-cellular feature extraction. Both of which are existing challenges with automated analysis systems.

There is an extensive literature on cell segmentation^8–17^ and tracking^18,19^. In^9–11,15^ morphological operations are first used to denoise the images followed by watershed or level set segmentation methods to get the final cell segmentation. In^17^, the nuclei information is provided for accurate cell segmentation. However, these methods do not provide accurate localization of the cell wall features with only cell boundary information that are needed for quantification. In^8,13,14^, they focus on improving the cell boundary segmentation accuracy. In^13,14^, they treat the cell segmentation problem as a semantic segmentation problem, using Generative Adversarial Networks (GAN) to differentiate between boundary pixels, cell interior, and background. These methods provide respectable accuracy on cell boundaries but they are not guaranteed to give a closed cell surface. The method proposed in^16^ can provide closed 2D surface while maintaining good 2D cell segmentation boundary results. It is challenging to do the downstream cell analysis such as cell tracking without a closed 3D cell surface. Based on segmentation or detection of cells^18,19^, rely on Viterbi algorithm to track cells. They require the global optimization which is inefficient to get the cell trajectory.

This paper presents a robust, time-lapse cell analysis method building upon our earlier work^8^. In^8^ we use Conditional Random Field (CRF) to get the improved cell boundaries while maintaining a closed cell surface. To make the segmentation method more robust to different datasets, we propose a modification to^8^ that incorporates rotation invariance in the 3D convolution kernels. A segmentation map labeling each individual cell in the 3D stack is thus created and a cell adjacency graph is constructed from this map. The adjacency graph is an undirected weighted graph with each vertex representing a cell and the weight on the edge representing the minimum distance between two cells. Based on this adjacency graph, sub-cellular features illustrated in Fig. 1 are computed. The cells are tracked by comparing the corresponding adjacency graphs in the time sequence similar to our previous work^20^. Details of the complete workflow will be described in section 3.

We demonstrate the performance of the proposed *segmentation* method on multiple cell wall tagged data sets. To demonstrate generality of our *tracking* method, we apply our *tracking* method on both cell wall tagged and nuclei tagged imagery. Compared to our previous work^8^, tracking and sub-cellular feature extraction are new problems considered. This paper additionally proposes a novel cell segmentation network architecture, using 3D rotation-equivariant layers. This paper also contains more experimental results of segmentation, tracking, and sub-cellular feature extraction.

In summary, the main contributions of this paper include:

- The first deep learning enabled end-to-end fully automated time-lapse 3D cell analysis method.
- A new 3D cell segmentation network with rotation equivariance that is robust to different imaging conditions.
- A novel graph based method for multiple instance tracking and sub-cellular feature extraction as well as the novel evaluation metrics to evaluate sub-cellular feature extraction accuracy.
- We will release a new membrane tagged imagery with partially (expert) annotated sub-cellular features and fully annotated by our computational method.

## Method

Our cell analysis method is illustrated in Fig. 1. First, we segment cells from each image stack in the time sequence. Second, the adjacency graph is built based on segmented images and is used to compute sub-cellular features and cell tracking features. Finally, quantitative measurements of the cell segmentation (cell wall, cell count, cell shape), sub-cellular features (junctions of three cell walls detection accuracy, anticlinal wall segment shape), and tracking results are provided.

### Segmentation

**Figure 2:**
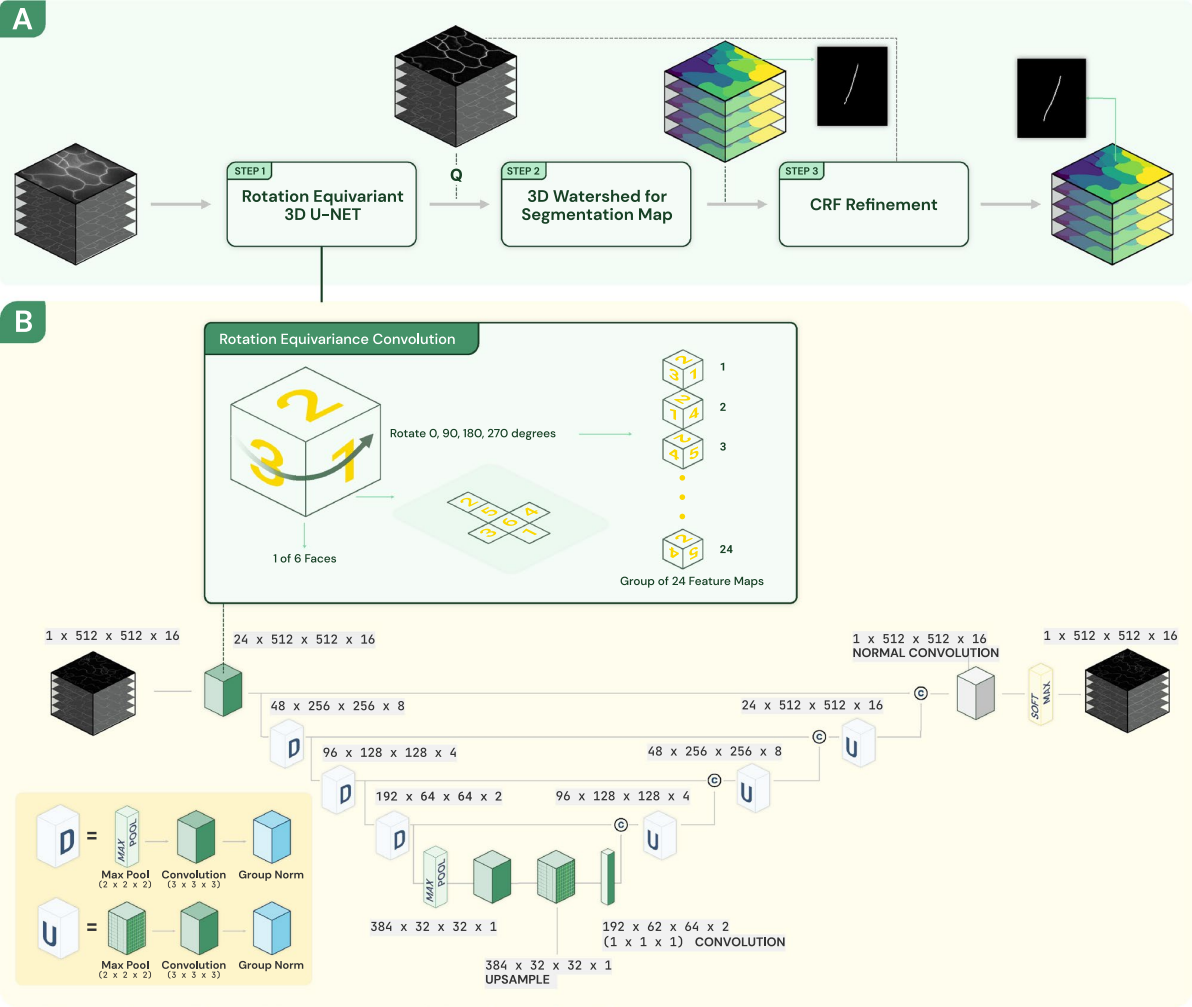
(**A**) Segmentation workflow includes rotation equivariant 3D U-Net, 3D watershed, and CRF refinement. (**B**) In 3D equivariant U-Net, all convolution layers are rotation equivariant convolution layers. The raw 3D image stack is truncated into 16 slices and then input to 3D equivariant U-Net.

We adopt the cell segmentation workflow from^8^ with rotation equivariance constrained enforced as shown in Fig. 2. 3D U-Net is a reliable method for semantic segmentation specifically for biomedical images, and 2D rotation equivariance has shown its robustness to input image orientation^21^. Therefore, we first use a rotation equivariance 3D U-Net to generate a probability map of each voxel being a cell wall. The full 3D U-Net rotation equivariance is achieved by replacing all convolution layers with rotation-equivariant layers described in the next paragraph. Second, to make sure we can get closed cell surfaces, a 3D watershed algorithm whose seeds are generated automatically is applied to the cell wall probability map, and outputs the initial cell segmentation result. The initial cell segmentation boundary is closed but may not be smooth because watershed segmentation is sensitive to noise. Finally, a conditional random field (CRF) model is used to refine the cell boundaries of the initial cell segmentation. The CRF model takes the cell wall probability map and initial cell segmentation labels as input and outputs a smooth and closed cell wall. In the following section, we will discuss the details of our rotation-equivariant convolution layers and the use CRF to refine the cell segmentation boundary.

3D rotation-equivariant layers are a generalization of convolution layers and are equivariant under general symmetry groups, such as the group of four 90° 2D rotations^21^. The corresponding 3D rotation group has 24 rotations as illustrated in Fig. 2 (A cube has 6 faces and any of those 6 faces can be moved to the bottom, and then this bottom face can be rotated into 4 different positions). To achieve this, convolution operations on feature maps are operating on a group of features which implies that we should have feature channels in groups of 24, corresponding to 24 rotations in the group.

For a given cell wall probability map **Q** and cell labels **X**, the conditional random field is modeled by the Gibbs distribution,

1 $$\begin{aligned} P({{\textbf {X}}}|{{\textbf {Q}}})=\frac{1}{Z({{\textbf {Q}}})}\exp (-E({{\textbf {X}}}|{{\textbf {Q}}})) \end{aligned}$$

where denominator $Z({{\textbf {Q}}})$ is the normalization factor. The exponent is the Gibbs energy function and we need to minimize the energy function $E({{\textbf {X}}})$ to get the final refined label assignments (for notation convenience, all conditioning is omitted from this point for the rest of the paper). In the dense CRF model, the energy function is defined as

2 $$\begin{aligned} E({{\textbf {X}}})=\sum _i\psi _u(x_i)+\sum _{i<j}\psi _p(x_i,x_j) \end{aligned}$$

where *i* and *j* are the indices of each voxel which iterate over all voxels in the graph, and $x_i$ and $x_j$ are the cell labels of vertices *i* and *j*. $i,j\in \{ 1,2, \ldots ,N \}$ and *N* is the total number of voxels in the image stack. $x_i,x_j\in \{ 0,1,2, \ldots ,L \}$ and *L* is the total number of cells identified by the watershed method (0 is the background class). The first term of Eq. (2), the unary potential, is used to measure the cost of labeling $i_{th}$ voxel as $x_i$ and it is given by $\psi _u(x_i)=-\log {P(x_i)},$ where $P(x_i)$ is the probability of voxel *i* having the label $x_i$. It is initially calculated based on the cell wall probability map **Q** and the label image of the watershed ${{\textbf {X}}}^0$ (The superscript 0 is used to denote the initial cell label assignment after watershed). $P(x_i^0)=1-q_i$ if voxel *i* is inside the cell with label $x_i^0$ after the watershed or if $x_i^0$ is the background label, and $P(x_i^0)=0$ otherwise. $q_i$ is the $i_{th}$ voxel value in the probability map from the rotation equivariant 3D U-Net. $1-q_i$ represents the probability of voxel being the interior point of the cell. The pairwise potential in Eq. (2) takes into account the label of neighborhood voxels to make sure the segmentation label is closed and the boundary is smooth^22^. It is given by:

3 $$\begin{aligned} \psi _p(x_i,x_j)=\mu (x_i,x_j)\sum _mw^{(m)}k^{(m)}({{\textbf {f}}}_i,{{\textbf {f}}}_j) \end{aligned}$$

where the penalty term $\mu (x_i,x_j)=1$ if $x_i\ne x_j$, and $\mu (x_i,x_j)=0$ otherwise. $w^{(m)}$ is the weight for each segmentation label $m\in \{0,1,2, \ldots ,L\}$, and $k^{(m)}$ is the pairwise kernel term for each pair of voxels *i* and *j* in the image stack regardless of their distance that capture the long-distance voxel dependence in the image stack. ${{\textbf {f}}}_i$ and ${{\textbf {f}}}_j$ are feature vectors from the probability map **Q**. ${{\textbf {f}}}_i$ incorporates location information of voxel *i* and the corresponding value in the probability map: ${{\textbf {f}}}_i = <{{\textbf {p}}}_i,q_i>$ where ${{\textbf {p}}}_i = <x_i, y_i, z_i>$, and $x_i, y_i$ and $z_i$ are the voxel *i* in the normalized coordinates in the range [0, 1]. Specifically, the kernel $k({{\textbf {f}}}_i,{{\textbf {f}}}_j)$ is defined as

4 $$\begin{aligned} k({{\textbf {f}}}_i,{{\textbf {f}}}_j)=\gamma _1\exp \left(-\frac{||{{\textbf {p}}}_i-{{\textbf {p}}}_j||^2}{2\sigma ^2_\alpha }-\frac{||q_i-q_j||^2}{2\sigma ^2_\beta }\right)+\gamma _2\exp \left(-\frac{||{{\textbf {p}}}_i-{{\textbf {p}}}_j||^2}{2\sigma ^2_\gamma }\right) \end{aligned}$$

where the first term depends on voxel location and the corresponding voxel value in probability map. The second term only depends on the voxel location. $\sigma _\alpha$, $\sigma _\beta$, $\sigma _\gamma$, $\gamma _1$, and $\gamma _2$ are the hyperparameters in Eq. (4). Based on our experiments, we have empirically chosen $\sigma _\alpha =3$ and $\sigma _\beta =5$, as these values work over a wide range of experimental data. These two hyperparameters control the degree of nearness and similarity of the probability map within a segmented region. $\sigma _\gamma$ is determined by the smallest possible segmentation region (cell size) allowed. $\gamma _1$, and $\gamma _2$ are weights for the loss function. The detailed explanations of each hyperparameter can be found in^22^. Finally, we pick the best label assignment ${{\textbf {X}}}^*$ as the final cell segmentation that minimizes energy function $E({{\textbf {X}}})$. The efficient CRF inference algorithm described in^22^ is used to find ${{\textbf {X}}}^*$ which is the final cell segmentation mask. In our experiments, $\sigma _\gamma$ is set to be 10, and $\gamma _1$, $\gamma _2$ are set to be 1 and 1.

### Tracking and feature computation

**Figure 3:**
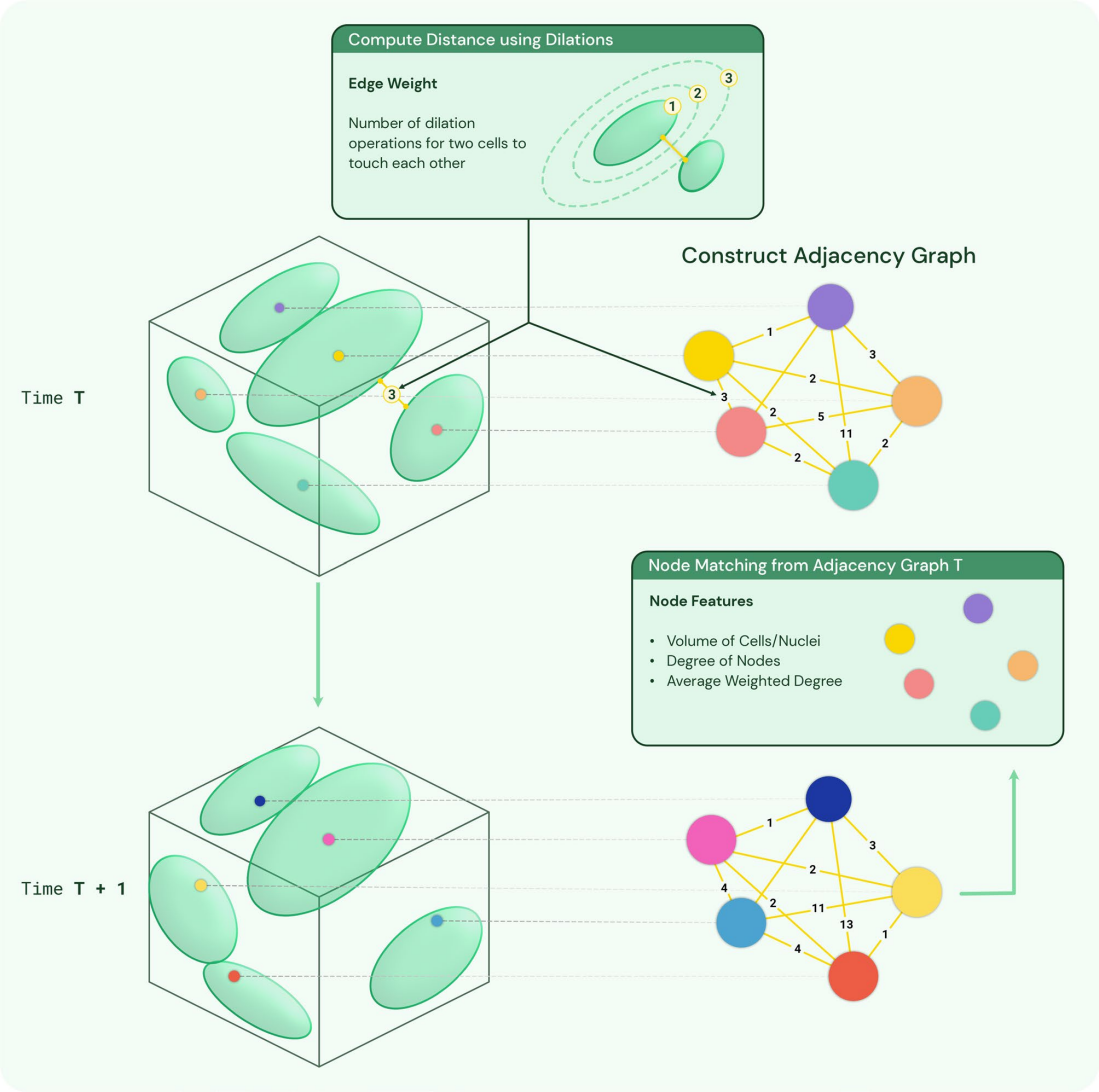
Constructing adjacency graph from the segmentation image and tracking cells/nuclei in consecutive frames using adjacency graph node features. Color of nodes denote the label/track of the cell/nuclei. Initially, random labels are assigned for each node in the adjacency graph. For T + 1 frame, after node matching for time T, track IDs are assigned to each node in T + 1.

After segmentation of 3D image stacks, the cells are detected and labeled in 3D space. Next, we utilize 3D spatial location of cells to build the adjacency graph for sub-cellular feature extraction and tracking as illustrated in Fig. 3.

Adjacency graph *G*(*V*, *E*) is a weighted undirected graph. The vertex $v_i \in V$ represents the $i-$th cell. For each pair of vertices $(v_i,v_j)$, there is an edge $e_i \in E$ connecting them. The weight $w_i \in W$ of the edge $e_i$ is the distance between cell *i* and *j*. The distance between two cells is computed as the number of morphology dilation operations needed of cells *i* and *j* until cell *i* and *j* become a single connected component. The details of this adjacency graph construction are given in Algorithm 1 below.
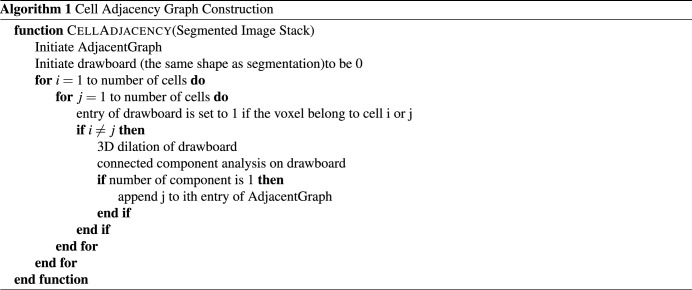


#### Sub-cellular feature extraction using adjacency graph

Sub-cellular feature extraction is based on the graph representation of the segmented image. To compute the anticlinal wall segments of cell *i*, we find all neighbor cells of cell *i*. The neighbor cells ${{\textbf {N}}}_i$ are defined to be all cells that are at a distance 1 from cell *i*. The anticlinal wall segments is found by collecting all points in the segmentation image shared by cell *i* and cell *j* where cell $j \in {{\textbf {N}}}_i$. To compute the junctions of 3 cell walls, we first pick cell $j \in {{\textbf {N}}}_i$. Then the junctions of 3 cell walls is computed as the points in the segmentation image shared by cell *i*, cell *j*, and cell *k* where cell *k* is ${{\textbf {N}}}_i \cap {{\textbf {N}}}_j$.

#### Tracking using adjacency graph

The assumption we make for the cell tracking is that in consecutive image stacks, cells should have similar relative location. For this, we will focus on computing features ${\textbf{f}}_{loc}$ that represent cell relative location information derived from the adjacency graph.

Cell location feature vector ${\textbf{f}}_{loc}$ is a two dimensional vector (*N*, *D*), where *N* is the total number of neighbor cells and *D* is the average distance from all other cells. Consider the adjacency graph *G*(*V*, *E*) of the segmented image stack. For node *i* in the graph, the location feature vector can be expressed as:

5 $$\begin{aligned} {\textbf{f}}_{loc}^i=(N,D)=(deg(v_i),\text {wdeg}(v_i)) \end{aligned}$$

where $v_i \in V$, $deg(v_i)$ is the cardinality of $N_i$, and $\text {wdeg}(v_i)$ is the weighted degree of the vertex $v_i$. The weighted degree of the vertex $v_i$ is defined as:

6 $$\begin{aligned} \text {wdeg}(v_i)=\frac{\sum _{j} w_{ij}}{\text {degree}(v_i)} \end{aligned}$$

where $\text {degree}(v_i)$ represents the degree of the vertex $v_i$. Then we compute the cell size by counting number of voxels within the cell. Combining the cell location feature and cell size feature, we get the three dimensional feature vector ${\textbf{f}}_{\text {track}}$. The details of algorithm used for calculating ${\textbf{f}}_{\text {track}}$ is described below.
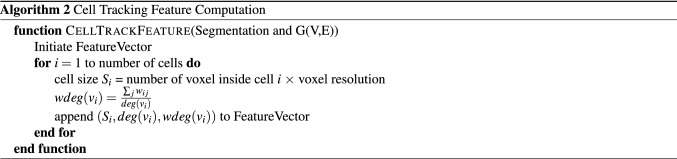


After computing ${\textbf{f}}_{track}^i$ for all nodes in two consecutive frames, we link two nodes from different frames based on the following similarity measurement *sim*(*i*, *j*) defined as

7 $$\begin{aligned}  sim(i,j) = \frac{|S_{1i}-S_{2j}|}{S_{1i}}+\frac{|deg_1(v_i)-deg_2(v_j)|}{deg_1(v_i)}+ \frac{|\text {wdeg}_1(v_i)-\text {wdeg}_2(v_j)|}{\text {wdeg}_1(v_i)} \end{aligned} $$

where *i* and *j* denote two nodes from two consecutive frames. We define *sim* so that we can allow different units of entries in ${\textbf{f}}_{\text {track}}^i$. We find $i^{*}$ and $j^{*}$ that minimizes *sim*. $i^{*}$ and $j^{*}$ are linked only when their *sim* is below a set threshold value. In our experiments, the threshold we use is between 0.1 and 0.5.

## Dataset

**Table 1:** Datasets summary and usage (note that TRA definition will be described in “Results” section).

Dataset	Dataset source	Brief description	Segmentation evaluation	Sub-cellular feature extraction evaluation	Tracking evaluation
Dataset 1	Membrane-tagged confocal single layer pavement cells	5 time sequences, each sequence has 9–20 image stacks, and each stack with 18–30 slices	Cell count and cell shape as evaluation metrics	Sub-cellular feature extraction results are provided	Full annotation is unavailable, so TRA score is not provided
Dataset 2	Membrane-tagged confocal multi layer pavement cells	6 time sequences, each sequence has 20 image stacks, and each stack with 119–139 slices	Segmentation boundary evaluation metrics	Sub-cellular feature annotations are not available, so evaluation is not possible	TRA evaluation metric is provided
Dataset 3	Nuclei-tagged *C. elegans* dataset	4 time sequences, each sequence has 140–250 image stacks, and each stack with 31–35 slices	not applicable	not applicable	TRA score provided

There are three datasets used in this paper. We use different evaluation for different datasets based on these datasets’ imaging subjects and annotations. Table 1 summarizes the datasets and their usage in this paper.

### Plasma-membrane tagged dataset

**Table 2:** Single layer pavement cell dataset^7^.

Dataset	Number of Time points	Image stack dimension (voxels)
Sequence 1	20	$512\times 512\times 20$
Sequence 2	9	$512\times 512\times 18$
Sequence 3	10	$512\times 512\times 30$
Sequence 4	13	$512\times 512\times 21$
Sequence 5	20	$512\times 512\times 25$

It consists of a long-term time-lapse from *A. thaliana’s* leaf epidermal tissue that spans over a 12 h period with a xy-resolution of 0.212 μm and 0.5 μm thick optical sections. The time step is 2 h for sequence#2 and is one hour for all other sequences. Anticlinal cell walls are partially annotated for all sequences. In addition to that, cells are partially annotated for sequence 5.

Two 3D confocal image stack datasets of fluorescent-tagged plasma-membrane cells are used in this paper. In both datasets, only the plasma-membrane signal is used and is represented by voxels with high intensity values. The first dataset^7^ (Dataset 1) consists of a long-term time-lapse from *A. thaliana’s* leaf epidermal tissue that spans over a 12 h period with a xy-resolution of 0.212 μm and 0.5 μm thick optical sections. There are 5 sequences of image stacks. Each sequence has 9-20 image stacks and each stack has 18 to 25 slices containing one layer of cells, and the dimension of each slice is $512\times 512$. Partial ground truth sub-cellular features are provided for this dataset. Details of this dataset are described in Table 2.

**Table 3:** Multi layer pavement cell dataset^23^.

Dataset	Image stack dimension (voxels)
Sequence 1	$512\times 512\times 134$
Sequence 2	$512\times 512\times 219$
Sequence 3	$512\times 512\times 119$
Sequence 4	$512\times 512\times 129$
Sequence 5	$512\times 512\times 139$
Sequence 6	$512\times 512\times 134$

It contains three layers of cell walls in the shoot apical meristem of *A. thaliana’s* that spans over 80 h with with a xy-resolution of 0.22 μm and 0.26 μm thick optical sections. The time step is 4 h for all sequences and each sequence has 20 frames. Cells with track IDs are fully provided.

The second dataset (Dataset 2) contains cells in the shoot apical meristem of 6 *Arabidopsis thaliana*^23^. There are 6 image sequences. Each image sequence has 20 image stacks. In each image stack, there are 129 to 219 slices containing of 3 layers (*L*) of cells: outer layer ($L_1$), middle layer ($L_2$), and deep layer ($L_3$), and the dimension of each slice is $512\times 512$. The available resolution of each image in x and y direction are 0.22 μm and in z is about 0.26 μm. The ground truth voxel-wise cell labels are provided, and each cell has a unique label. Each cell track also has a unique track ID. Details of this dataset are described in Table 3.

### Cell nuclei dataset

**Table 4:** *C. elegans* developing embryo nuclei dataset^31,32^.

Dataset	Time step (min)	Number of frames	Image stack dimension (voxels)
Sequence 1	1	250	$512\times 708\times 35$
Sequence 2	1.5	250	$512\times 712\times 31$
Sequence 3	1	190	$512\times 712\times 31$
Sequence 4	1.5	140	$512\times 712\times 31$

The resolution of each image stack is $0.09 \, \upmu m\times 0.09 \, \upmu m\times 1.0 \, \upmu m$. Sequence 1 and 2 are training set which contains partial nuclei segmentation with track IDs for training. Sequence 3 and 4 are testing set so no annotations available.

The 3D time-lapse video sequences of fluorescent nuclei microscopy image of *C. elegans* developing embryo (Dataset 3). Each voxel size is $0.09 \times 0.09 \times 1.0$ in microns. Time points were collected once per minute for five to six hours. There are two videos in the training set and two videos in the testing dataset. Details of this dataset are described in Table 4. This dataset is used to evaluate our tracking algorithm performance.

**Figure 4:**
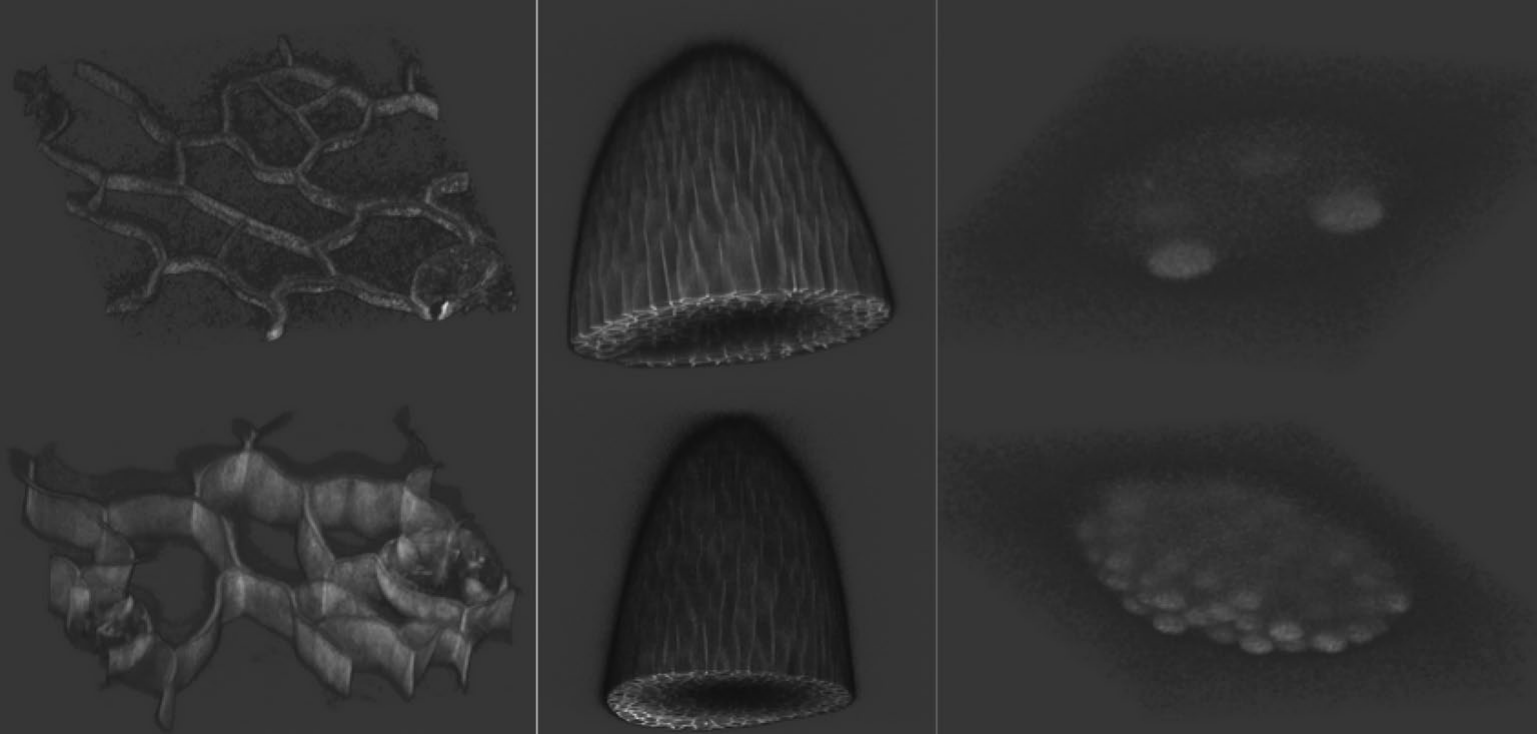
The figure shows two 3D example image stacks from each dataset. The left column is Dataset 1, mid column is Dataset 2, and right column is from Dataset 3.

3D visualization of three datasets are shown in Fig. 4.

## Results

### Segmentation

**Figure 5:**
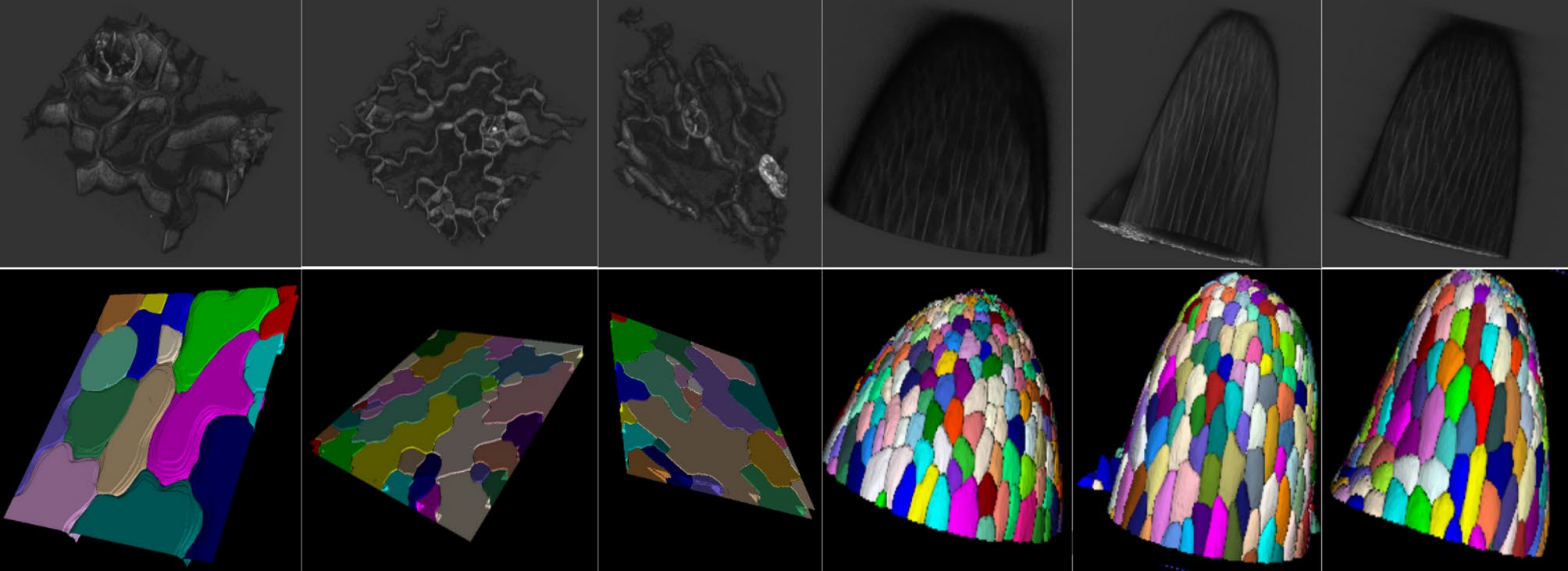
The figure shows three 3D segmentation image stacks. The top row is 3D view of confocal images, and bottom row is the 3D view of segmentation results. Left three samples are from Dataset 1 and right three samples are from Dataset 2.

**Table 5:** Cell counting accuracy for different methods.

Sequence	Ground truth	ACME^10^	MARS^11^	Supervoxel method^12^	Our method
Sequence 1	23	21.5 (3.2)	25.5 (2.2)	24 (1.1)	23.5 (0.9)
Sequence 2	30	41.1 (3.1)	35.1 (2.8)	32 (2.1)	30.1 (0.8)
Sequence 3	25	22.6 (2.1)	27.5 (3.2)	24 (1.5)	25 (0.5)
Sequence 4	18	18.8 (1.2)	18.5 (1.2)	18.2 (1.2)	18 (0.6)
Sequence 5	28	31.5 (2.9)	24.5 (2.3)	26.2 (1.1)	27.8 (1.0)

For each time sequence, there is a fixed number of cells. Due to segmentation error, the algorithms can generate different number of cells for different time points of the sequence. The table shows average number of detected cells (standard deviation values in parenthesis) for the entire sequence.

Since Dataset 1 does not have fully annotated 3D cell boundaries, we train all machine learning models using the entire Dataset 2. To evaluate different methods’ performance on Dataset 2, we use the following train/test split. We randomly divide the whole datasets into three folds (train, validation, and test). To evaluate segmentation performance on test fold, we train models on all image stacks (volumes) of other two folds. We use the validation set to pick the best trained model with smallest validation error. We use above strategy three times for each layer and compute average and standard deviation with respect to evaluation metrics in Table  6. 3D visualization of the segmentation results on Dataset 1 and 2 are shown in Fig. 5.

**Figure 6:**
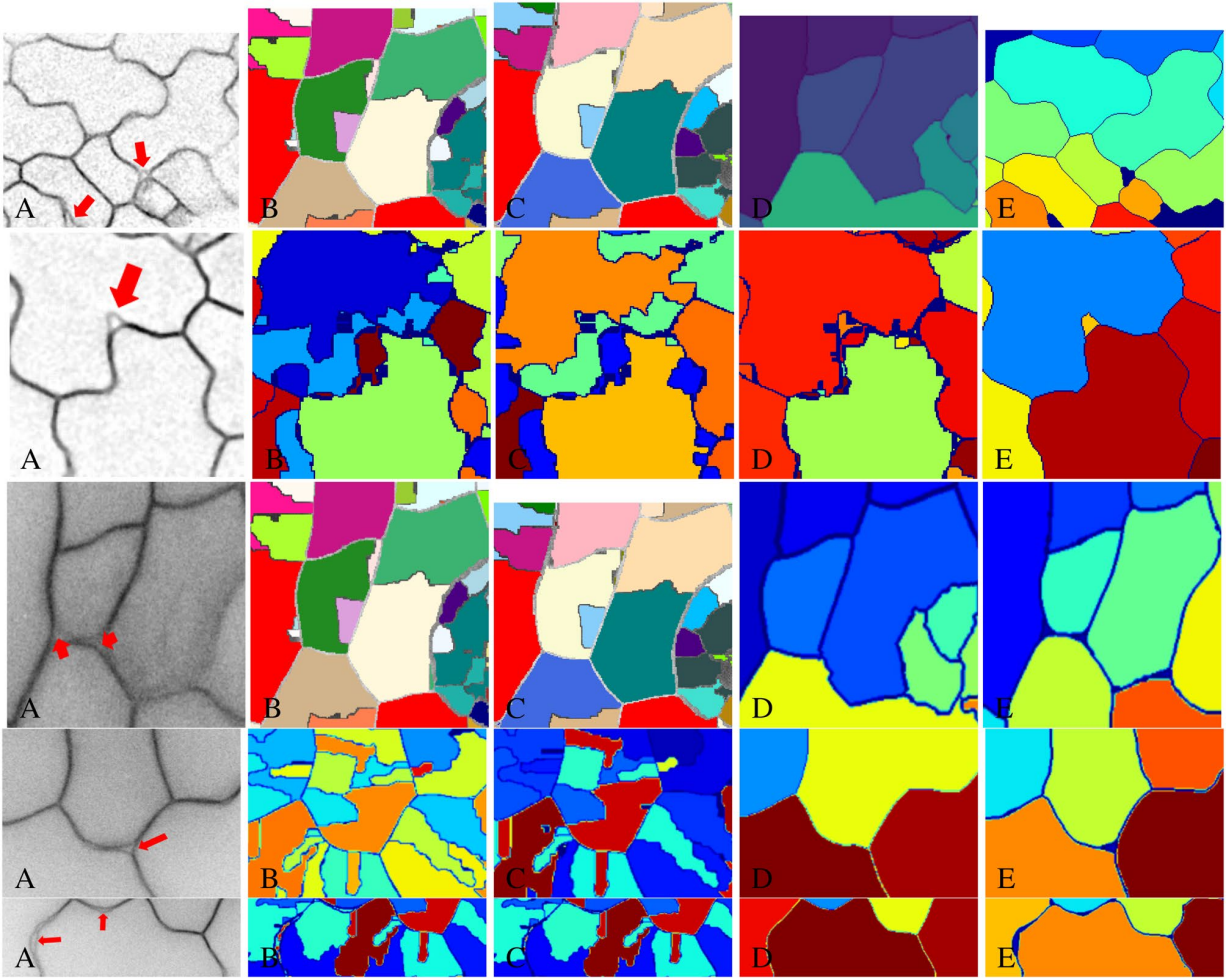
The figure shows the segmentation results of the cell image with inter-cellular space or *protrusion* indicated by a red arrow. (**A**) Inverted raw image in xy orientation, (**B**) MARS, (**C**) ACME, (**D**) supervoxel-based method, (**E**) proposed method.

We apply our proposed method to the Dataset 1 for the purpose of identifying and analyzing cells based on the segmentation. The segmentation results of our proposed method and other state-of-the-art methods are shown in Fig. 6. Our proposed method has visually better segmentation performance with closed cell surface and smooth boundary, and our method is able to identify the inter-cellular spaces and *protrusions* in the 3D cell image stack. For Dataset 1, we do not have full cell annotations, so we only evaluate the cell counting accuracy on this dataset.

**Table 6:** 3D segmentation performance on $L_1$, $L_2$, and $L_3$.

$L_1$	Precision	Recall	F-score
ACME^10^	0.805	0.966	0.878
MARS^11^	0.910	0.889	0.899
Supervoxel method^12^	**0.962**	0.932	0.947
Our method	0.961 (0.012)	**0.973** (0.012)	**0.967** (0.012)

$L_2$	Precision	Recall	F-score
ACME^10^	0.775	**0.980**	0.866
MARS^11^	0.921	0.879	0.900
Supervoxel method^12^	0.910	0.932	0.921
Our method	**0.955** (0.012)	0.971 (0.012)	**0.963** (0.012)

$L_3$	Precision	Recall	F-score
ACME^10^	0.745	**0.976**	0.845
MARS^11^	0.909	0.879	0.894
Supervoxel method^12^	**0.982**	0.881	0.929
Our method	0.955 (0.011)	0.942 (0.018)	**0.949** (0.013)

The bold values represent the best performing method for each of the metrics.

If there is a detected boundary voxel by algorithms within 5 voxels of a ground truth boundary voxel, then it is considered as a correct detection, otherwise it is considered as a miss detection. If there is a detected boundary voxel by algorithms within 5 voxels of a voxel that is not ground truth boundary voxel, then it is considered as a false detection. Numbers in brackets are standard deviation values.

For each sequence, there are a fixed number of cells for all time points. Therefore, we want segmentation algorithms to generate average cell counting results close to ground truth counting numbers, and the variance of counting results for one sequence should be as small as possible. Details of the cell counting results are in Table  5. Clearly, our method has the best cell counting performance.

**Figure 7:**
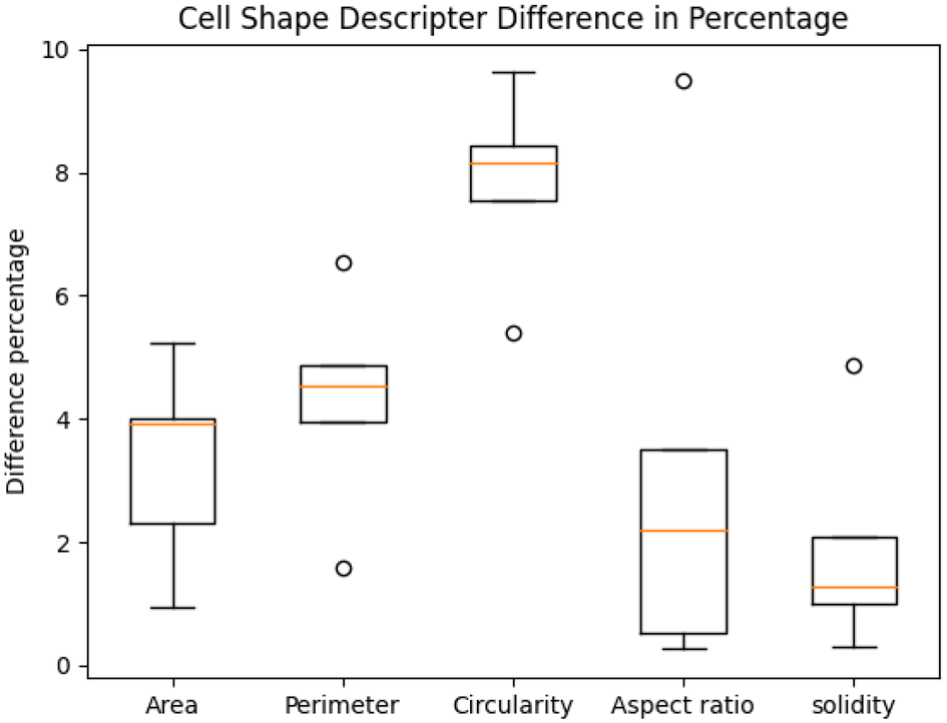
3D segmentation evaluation using cell shape descriptors including area, perimeter, circularity, aspect ratio, and solidity (ratio between cell area and its convex hull area). The difference is in terms of percentage.

In order to verify if the output of the segmentation can be used for time lapse sequence analysis, we calculate basic cell shape information from the maximum area plane of the cells to compare with the expert annotations. The maximum area plane of a cell is the image plane which has the largest cell area across all z-slices. The shape information includes area, perimeter, circularity, and solidarity. Figure 7 shows the comparison. Note that not all cells are annotated so that some cell comparisons are missed. The average shape difference is 4.5 percent and the largest shape difference is within 10 percent.

Next, we apply our cell segmentation method on Dataset 2. Boundary precision, recall, and F1 score are used to evaluate the boundary segmentation accuracy. Specifically, given a ground truth boundary image G and a computed boundary image B, we can define the following measurements:

- True positives (TP): Number of boundary pixels in G for which exist a boundary pixel in B in range R.
- False negatives (FN): Number of boundary pixels in G for which does not exist a boundary pixel in B in range R.
- False positives (FP): Number of boundary pixels in B for whose does not exist a boundary pixel in G in range R

Then boundary precision is defined to be: $\frac{TP}{(TP+FP)}$ and recall: $\frac{TP}{TP+FN}$.

In our experiment, we set R to be 5. Table 6 shows the comparison of the final segmentation boundary result using our proposed method and other methods including ACME^10^, MARS^11^ and a supervoxel-based algorithm^12^ on $L_1$ to $L_3$ respectively. In terms of cell wall accuracy, our model shows at least 0.03 improvement in the F-score measure on average in terms of cell wall segmentation accuracy.

It is noted that the average segmentation time of our proposed model is significantly shorter compared to the supervoxel-based method^12^. Our proposed method takes approximately 0.8 seconds to segment one 512 × 512 image slice on average, whereas supervoxel-based method takes approximately 6 seconds on a NVIDIA GTX Titan X with an Intel Xeon CPU E5-2696 v4 @ 2.20GHz. We have also integrated the proposed segmentation method into BisQue. There are three hyperparameters in the BisQue segmentation module. “Minimum Distance” is $\sigma _\gamma$ in Eq. (4). “Label Threshold” relates to the variation in the cell volumes within the datasets. This is used to ensure small regions such as protrusions are not labeled as cells. Intensity values below “Threshold” are ignored. “Threshold” is typically between 0 and 0.1 for a normalized image.

### Tracking and feature extraction

**Figure 8:**
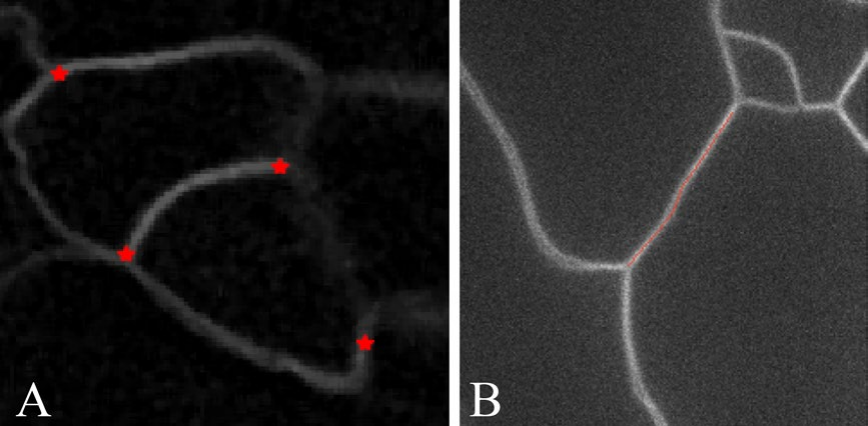
(**A**) Extracted junctions of three cell walls, (**B**) Extracted anticlinal wall segment.

We apply our whole workflow on Dataset 1 to extract sub-cellular features like anticlinal wall segments and junctions of 3 cell walls. Qualitative results of the extracted sub-cellular features are shown in Fig. 8.

**Figure 9:**
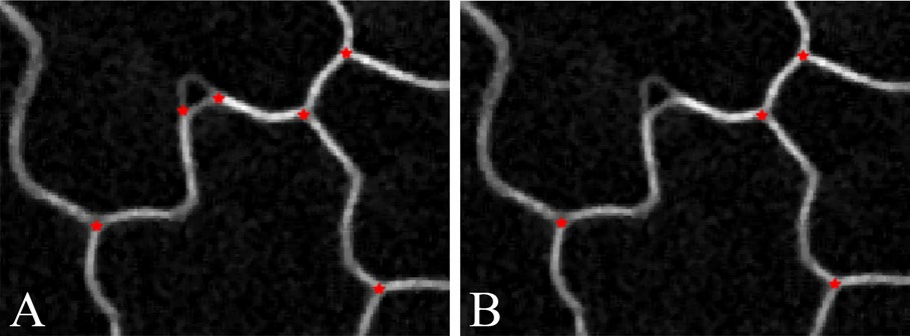
Example of computing 3 cell wall junctions from raw image (**A**), and (**B**) using our method.

**Table 7:** Quantitative analysis on error of junctions of three cell walls.

Algorithm	Precision	Recall	F1
Corner Detection^24^	0.893 (0.013)	0.962 (0.008)	0.926 (0.01)
ACME^10^	0.829 (0.015)	0.964 (0.009)	0.891 (0.011)
MARS^11^	0.823 (0.014)	**0.980** (0.006)	0.895 (0.009)
Supervoxel method^12^	0.933 (0.009)	0.911 (0.012)	0.922 (0.01)
Our Method	**0.980** (0.007)	0.945 (0.006)	**0.962** (0.006)

The bold values represent the best performing method for each of the metrics.

Precision, recall, and F1 score are used to evaluate the detection of those junctions. Number in parenthesis are standard deviation values across all images stacks.

The quantitative measurement of accuracy of junctions of 3 cell walls is also provided. We compare our results with 3D corner detection based method^24^ on the raw image stack, and applying our 3 cell wall junction detection method using the segmentation image from other state-of-the-art methods^10–12^. The 3 cell wall junction detection results are shown in Table 7. If 3 cell wall junctions are detected within 5 voxels of a ground truth 3 cell wall junction, it is a correct detection. Then we define false positive (FP), and false negative (FN) based on the binary detection of 3 cell walls junction. The error (E) is defined by the summation of FP and FN and normalized by total number of true 3 cell wall junctions. The results in the Table 7 are average values across all image stacks. From the table, we can see our method has the best 3 cell wall junction detection accuracy in terms of F1. Compared to the method that directly computes 3 cell wall junctions from raw image, our method has significantly better performance in terms of FP. This is because not all corner points are junctions of three cell walls. For example, corner detection based method gives false positive in the case shown in Fig. 9. Our graph based image feature extraction model not only uses low level image features but also some semantic information.

The anticlinal wall segment is defined by two neighboring junctions of 3 cell walls are also computed. The partial annotation of such segments are provided. We would like to note that such manual annotations are very labor intensive and it is impractical to annotate all anticlinal cell wall segments (see Fig. 1) even in a single 3D volume. The practical difficulties include lack of support for 3D visualization and annotation tools for tracing. The ground truth segments were annotated by going through each slice in the image stack, finding the approximate slice where neighboring cell walls touch, and then tracing the segment in that single slice. Each segment in the ground truth is represented by a collection of coordinates of the segment in that image slice. Note that different segments can be on different slices. In contrast, each of our computed segments can span multiple Z slices, hence providing a more accurate 3D representation than is manually feasible. This also makes it challenging to compare the manual ground truth with the computed results.

### Evaluation metrics for anticlinal wall segments

We propose a set of evaluation metrics for the detected anticlinal wall segments as there are no prior works on this topic.

1. *End-point Displacement error (EDE)* in the end points of the two segments. Given two segments *P* with *m* points and *Q* with *n* points, two end points of *P* are $p_1$ and $p_m$ and two end points of *Q* are $q_1$ and $q_n$. EDE is defined as
  8 $$\begin{aligned}  EDE(P,Q) = \frac{1}{2}(\Vert p_1-p_m\Vert +\Vert q_1-q_n\Vert ) \end{aligned} $$
  where $\Vert \cdot \Vert$ is $l^2$ norm.
2. *Fréchet distance (FD)*^25^ between the two segments. FD is a measure of shape similarity of two curves and it takes into account the location and ordering of points along the curves. Mathematically, consider two curves *P* with *m* points and *Q* with *n* points. *P* contains a sequence of points $(p_1, \ldots ,p_m)$ and *Q* contains a sequence of points $(q_1, \ldots ,q_n)$. A coupling *L* between *P* and *Q* is a sequence $(p_{a_1},q_{b_1}),(p_{a_2},q_{b_2}), \ldots ,(p_{a_z},q_{b_z})$ of distinct pairs from *P* and *Q* such that $a_1=1$, $b_1=1$, $a_z=m$, and $b_z=n$, and for all $i=1, \ldots ,z-1$ we have $a_{i+1}=a_i$ or $a_{i+1}=a_i+1$, and $b_{i+1}=b_i$ or $b_{i+1}=b_i+1$. Thus the order of those points are kept in the coupling *L*. The length $\Vert L\Vert$ of the coupling *L* is the length of the longest Euclidean distance in *L*:
  9 $$\begin{aligned}  \Vert L\Vert = \max _{i=1, \ldots ,z}d(p_{a_i},q_{b_i}) \end{aligned} $$
  where *d* is the Euclidean distance. Then FD *F* is defined as:
  10 $$\begin{aligned}  F(P,Q) = \min \{ \Vert L\Vert \} \end{aligned} $$
  where *L* is a coupling of *P* and *Q*.
3. Length difference (LD), absolute difference in lengths between the two segments.
4. Percentage difference in length (DP), length difference normalized by ground truth length.

**Figure 10:**
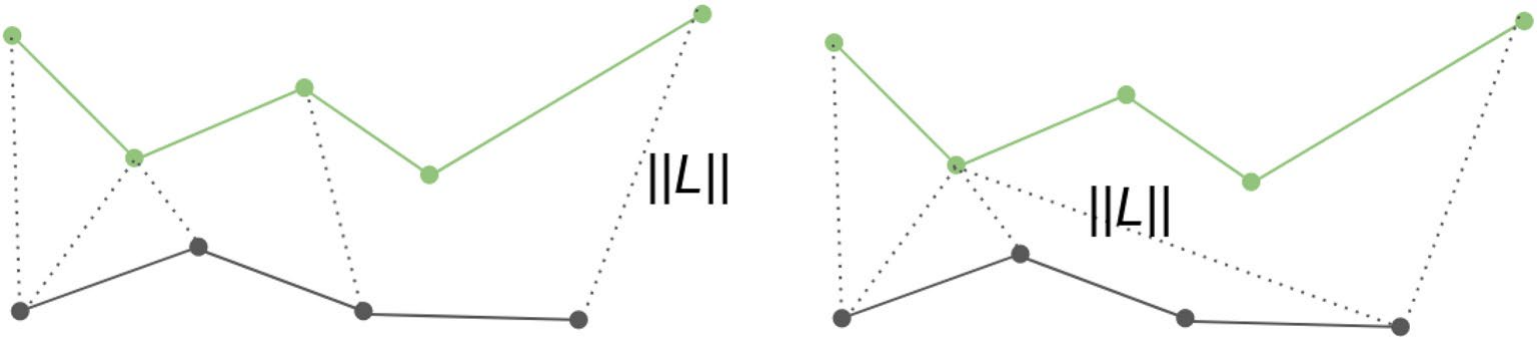
The figure shows two examples of coupling *L*. Dashed lines represent distinct pairs. $\Vert L\Vert$ is the length of the longest distance of those pairs. Finally, FD is the minimum of those $\Vert L\Vert$.

Figure 10 illustrates the definition of FD.

**Table 8:** Anticlinal cell wall segment evaluation on Dataset 1 using EDE, FD, LD, DP.

Sequence	EDE	FD	LD	DP
Sequence 1	2.8	4	2.32	2.9
Sequence 2	6.4	8.4	4.19	6.1
Sequence 3	3.05	3.9	1.74	2.4
Sequence 4	3.02	3.7	2.24	2.3
Sequence 5	2.33	3.4	2.11	2.1
Mean (standard deviation)	3.52 (1.64)	4.68 (2.09)	2.52 (0.96)	3.16 (1.67)

Average EDE between the results using our method and the ground truth is 3.03 voxels, average FD is 3.7 voxels, average LD is 2.24 voxels, and average DP is 2.3 percent. Evaluation results of different time series are shown in Table 8 and evaluation result for each segment is in the supplemental materials.

**Table 9:** Cell tracking performance on dataset 2 and dataset 3 using TRA.

Dataset 2	Viterbi tracker^18^	Cell proposal^33^	Our method
Sequence 1	0.513	0.492	**0.571**
Sequence 2	0.520	0.512	**0.593**
Sequence 3	0.488	0.532	**0.581**
Sequence 4	0.533	0.498	**0.566**
Sequence 5	0.542	0.525	**0.602**
Sequence 6	0.518	0.542	**0.544**
Mean (standard deviation)	0.519 (0.019)	0.517 (0.020)	**0.576** (0.020)

Dataset 3	KIT-Sch-GE^27^	KTH-SE^28^	Our method
Sequence 1	0.903	**0.942**	0.931
Sequence 2	0.906	0.893	**0.912**
Sequence 3 and 4	0.886	**0.945**	0.895

The bold values represent the best performing method for each of the metrics.

We also apply our tracking method on Dataset 2 and Dataset 3. Table 9 shows the quantitative comparison of our method with other state-of-the-art cell/nuclei tracking methods. The evaluation metric we use is tracking accuracy (TRA), proposed in^26^. TRA measures how accurately each cell/nuclei is identified and followed in successive image stacks of the sequence. Ground truth tracking results and tracking results generated from algorithms are viewed as two acyclic oriented graphs and TRA measures the number of operations needed to modify one graph to another. More specifically, TRA is defined on Acyclic Oriented Graph Matching (AOGM) as

11 $$\begin{aligned} \text {TRA} = 1 - \dfrac{\min (\text {AOGM}, \text {AOGM}_0)}{\text {AOGM}_0} \end{aligned}$$

where AOGM$_0$ is the AOGM value required for creating the reference graph from scratch. TRA ranges between 0 to 1 (1 means perfect tracking). Our method shows a rough 0.05 TRA measurement improvement on Dataset 2. To demonstrate the robustness of our tracking method, we also apply it on Dataset 3, a cell nuclei dataset, and achieve a TRA of 0.895 which is comparable to state-of-the-art tracking methods on IEEE ISBI CTC2020 cell tracking challenge. State-of-the-art methods^27,28^ are based on the traditional Viterbi cell tracking algorithm whose complexity is ${\mathcal {O}}(TM^{4})$ where *T* is the length of the sequence and *M* is the maximum number of cells/nuclei. In contrast, the complexity of our method is ${\mathcal {O}}(TM^{2})$. Sequence 1 and 2 are the training data released from the challenge and we run the state-of-the-art methods on the individual sequence to get TRA evaluation metric. Sequence 3 and 4 are testing data that is not published by the challenge and TRA values are given by the challenge organization.

In summary, we did extensive experiments and used different evaluation metrics to demonstrate the performance of our method. For segmentation, we use cell counting accuracy in Table  5, cell shape evaluation metric Fig. 7, and cell boundary segmentation accuracy in Table 6 to show the performance of our method. For sub-cellular feature extraction, we use precision, recall, and F1 score as in Table  7 to evaluate 3 cell wall junctions detection performance, and we use EDE, FD, LD, and DP in Table  8 to evaluate the segments detection performance. For tracking, we use TRA in Table 9 as the evaluation metric.

## Conclusion

In this paper, we present an end-to-end workflow for extracting quantitative information from 3D time-lapse imagery. The workflow includes 3D segmentation, tracking, and sub-cellular feature extraction. The 3D segmentation pipeline utilizes deep learning models with rotation equivariance. Then an adjacency graph is built for cell tracking and sub-cellular feature extraction. We demonstrate the performance of our model on multiple cell/nuclei datasets. In addition, we also curate a new pavement cell dataset with partial expert annotations that will be made available to researchers.

The proposed segmentation method is implemented as a computational module in BisQue^29,30^. Users can run the CellECT2.0 module using the following steps: (1) Navigate to BisQue on their web browser and create an account, (2) Upload their own data in TIFF format or use suggested example dataset, (3) Select an uploaded TIFF image or use our example, (4) Set hyper parameters of the module (default value to run Dataset 1) and select Run and the BisQue service will compute the segmentation results and display it in the browser. The runtime for a $512 \times 512 \times 18$ image is approximately one minute using a CPU node with a 24 core Xeon processor and 128GB of RAM. We provide screenshots of these steps in the Supplemental Materials.

## Supplementary Information

Supplementary Information 1.

Supplementary Information 2.

## Data Availability

The code is available on GitHub. Dataset 1, Dataset 2, and Dataset 3 analyzed during the paper are available in the repository, Dataset 1, Dataset 2, and Dataset 3 separately.
