# Supplementary material for: Segmentation, tracking, and sub-cellular feature extraction in 3D time-lapse images
Source: Sci Rep. 2023 Mar 1;13:3483. doi: 10.1038/s41598-023-29149-z (PMC9977871; doi:10.1038/s41598-023-29149-z)
Supplement: Supplementary file 2 — Supplementary Information 2. [file 41598_2023_29149_MOESM2_ESM.pdf]

Supplemental Materials

I. Segmentation and sub-cellular feature extraction from BisQue

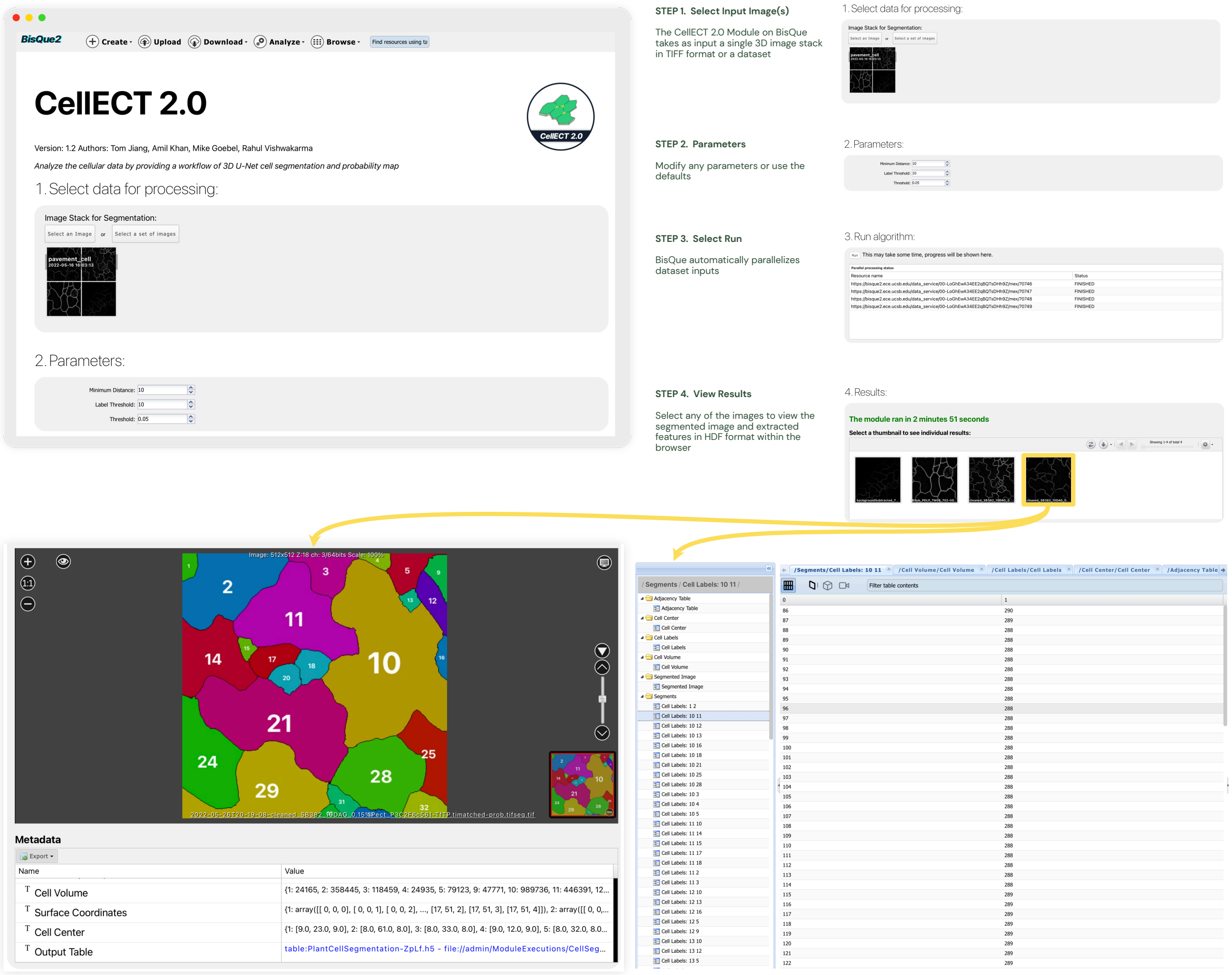

Fig. 1. **CellECT2.0 Module in BisQue.** Here we have an example of four 3D stacked images from different time sequences of Dataset 1 in TIFF format as input to our module. BisQue will automatically parallel process the input images and return the results for each image. Each segmented cell is labeled with an integer in the segmentation image and each of the outputs have an associated HDF file containing the cellular and sub-cellular features, as summarized in Table I.

Cellular and sub-cellular features provided by BisQue are summarized in table I.

The full cellular and sub-cellular results for this image stack can be accessed here.

TABLE I
CELLULAR AND SUB-CELLULAR FEATURES PROVIDED BY BISQUE

**Different features**	**Example Values or Explanation**
Cell Volume	number of voxels inside a cell, for example, 358,445
Neighboring (Adjacent) cells	for example, cells 1,3,11,14 are neighboring cells of cell 2
3D cell surface	list of coordinates of surface of points for one cell
Three cell wall junction points	for example, junction point of cell 2,3,11 is (12,207)
Cell Center	for example, center of cell 2 is (8,61,8)
Segments	list of coordinates of points along that segment

II. SEGMENTS EVALUATION

Some examples of segments evaluation are summarized in Table II. The evaluation metrics include: Euclidean error of start (end) points of segments, Fréchet Distance, and length difference of segments. In Table II, the first column is segment ID. Segment ID includes two parts: Sequence ID and the segment ID within the sequence. Sequence ID starts with "LGMTPM" and ends with a 2-digit number to differentiate different sequences. Segment ID contains information about which frame this segment comes from. For example, "LGMTPM01 Seg03T28" means this segment comes from frame 28 of sequence 01.

Detailed comparison results are in the `segments.xlsx` file.

TABLE II
SEGMENTS EVALUATION RESULTS. THE RESULTS INCLUDE SEGMENTS' END POINTS LOCATION ACCURACY, SEGMENTS LENGTH ACCURACY, AND FRECHET DISTANCE FOR SEGMENTS SHAPE ACCURACY

	detected start point x	detected start point y	detected start point z	detected end point x	detected end point y	detected end point z	GT start point x	GT start point y	GT end point x	GT end point y	euclidean distance error start point	euclidean distance error end point	Frechet Distance	GT Length	Detected Length	difference	% change
LGMTPM01 Seg03T28	391	215	3	485	203	3	392.7	216.2	481.8	203.3	2.0	3.2	3.9	95.1	93.4	1.7	1.8
LGMTPM01 Seg06T24	121	144	4	195	142	5	124.9	145.3	196.0	139.0	4.1	3.2	4.1	85.6	87.2	1.6	1.9
LGMTPM01 Seg02T01	169	356	2	234	360	4	172.8	358.3	233.3	360.3	4.5	0.7	4.5	66.6	70.3	3.6	5.4
LGMTPM01 Seg05T01	303	274	6	350	320	2	303.5	273.3	346.8	320.0	0.9	3.3	3.3	70.7	72.9	2.2	3.1
LGMTPM01 Seg08T01	200	59	1	299	82	2	204.3	59.8	297.3	81.8	4.3	1.8	4.3	100.6	98.1	2.4	2.4
LGMTPM02 Seg05T23	207	138	4	217	77	5	210.2	143.8	213.3	82.0	6.6	6.2	8.4	68.9	73.1	4.2	6.1
LGMTPM03 Seg02T32	178	212	4	246	183	4	182.7	212.3	243.7	182.7	4.7	2.4	4.1	77.8	80.3	2.5	3.2
LGMTPM04 Seg03T17	286	327	5	328	392	7	289.7	329.3	325.2	395.7	4.3	4.6	4.7	85.2	83.3	2.0	2.3
LGMTPM05 Seg03T01	45	220	3	145	224	5	48.8	219.3	145.3	220.3	3.8	3.8	3.7	106.5	108.1	1.6	1.5
LGMTPM05 Seg03T02	11	198	2	100	200	4	13.5	196.5	101.5	198.0	2.9	2.5	3.0	98.0	100.0	2.0	2.0
LGMTPM05 Seg03T01	12	201	3	100	200	5	13.0	202.5	102.3	203.5	1.8	4.2	2.9	99.3	97.0	2.2	2.3
LGMTPM05 Seg03T03	13	200	10	103	200	8	14.0	199.8	102.5	201.0	1.0	1.1	3.6	98.5	102.1	3.6	3.7
LGMTPM05 Seg03T04	17	208	9	102	211	7	15.5	205.5	104.5	206.3	2.9	5.4	3.7	99.0	103.1	4.1	4.1
LGMTPM05 Seg03T05	16	210	8	105	212	8	15.8	209.3	105.3	210.3	0.8	1.8	3.5	99.5	101.2	1.7	1.7
LGMTPM05 Seg03T06	18	184	6	105	185	8	17.0	182.5	106.8	184.0	1.8	2.0	3.2	95.8	98.1	2.3	2.4
LGMTPM05 Seg03T07	19	216	7	104	218	9	17.0	215.8	106.8	216.5	2.0	3.1	3.3	99.8	101.2	1.5	1.5
LGMTPM05 Seg03T08	18	221	6	105	221	8	17.0	220.0	106.5	220.8	1.4	1.5	3.6	99.6	102.3	2.7	2.7
LGMTPM05 Seg03T09	18	200	6	110	201	7	19.3	199.0	109.5	199.8	1.6	1.3	3.1	100.3	101.3	1.0	1.0
LGMTPM05 Seg03T10	24	192	5	110	195	7	22.0	193.5	112.3	194.0	2.5	2.5	3.3	100.3	102.3	2.1	2.0
LGMTPM05 Seg03T11	23	197	6	115	200	9	22.0	196.9	113.3	197.8	1.0	2.9	3.4	101.3	101.2	0.1	0.1
LGMTPM05 Seg03T12	25	200	11	117	199	9	25.0	198.5	115.3	198.3	1.5	1.8	3.3	100.3	101.4	1.0	1.0
LGMTPM05 Seg03T13	25	201	10	114	198	8	24.3	197.3	116.3	200.5	3.8	3.4	3.2	102.1	104.3	2.2	2.2
LGMTPM05 Seg03T14	27	200	9	118	200	7	25.5	198.5	118.0	200.3	2.1	0.3	3.6	102.5	103.3	0.7	0.7
LGMTPM05 Seg03T15	26	199	7	119	202	7	29.0	197.8	122.5	198.3	3.3	5.1	3.2	103.5	101.5	2.0	2.0
LGMTPM05 Seg03T16	29	195	11	122	199	12	31.3	196.3	125.0	197.8	2.6	3.3	3.4	103.8	100.3	3.5	3.3
LGMTPM05 Seg03T17	32	220	10	126	220	11	33.8	221.0	127.0	222.8	2.0	2.9	3.5	103.3	101.3	2.0	1.9
LGMTPM05 Seg03T18	16	230	9	113	230	10	17.0	229.3	111.8	230.8	1.3	1.5	3.7	104.8	106.4	1.6	1.5
LGMTPM05 Seg03T19	35	231	8	130	232	9	34.8	230.0	129.3	230.8	1.0	1.5	3.6	104.5	107.9	3.4	3.2
LGMTPM05 Seg03T20	37	220	9	130	224	10	37.5	221.3	132.3	222.3	1.3	2.9	3.9	104.8	106.8	2.0	1.9
LGMTPM05 Seg03T21	31	226	8	129	230	8	30.5	225.5	126.5	227.0	0.7	3.9	3.1	106.0	101.3	4.7	4.4
LGMTPM05 Seg03T22	47	220	9	144	223	10	48.8	219.3	145.3	220.3	1.9	3.0	4.0	106.5	105.9	0.6	0.6
LGMTPM04 Seg01T01	365	360	12	433	425	13	366.0	358.0	429.3	424.0	2.2	3.9	3.1	101.4	99.3	2.2	2.1
LGMTPM04 Seg01T02	366	362	13	430	427	14	365.8	362.5	429.0	429.5	0.6	2.7	3.2	102.1	104.0	1.8	1.8
LGMTPM04 Seg01T03	365	370	12	428	436	12	368.0	371.0	431.3	438.0	3.2	3.8	3.2	102.1	104.7	2.5	2.5
LGMTPM04 Seg01T04	367	378	11	429	449	11	367.3	379.3	431.5	445.3	1.3	4.5	4.0	102.1	101.2	0.9	0.9
LGMTPM04 Seg01T05	365	381	12	435	448	13	367.8	383.3	431.3	450.5	3.6	4.5	4.2	103.5	100.9	2.6	2.5
LGMTPM04 Seg01T06	363	375	14	429	453	15	368.3	380.0	433.0	450.0	7.3	5.0	3.6	105.4	102.3	3.0	2.9
LGMTPM04 Seg01T07	369	385	11	433	450	12	369.3	385.5	430.8	450.3	0.6	2.3	3.4	99.3	98.5	0.8	0.8
LGMTPM04 Seg01T08	370	381	11	432	455	11	370.3	385.3	432.8	451.1	4.3	4.0	3.1	100.7	103.7	3.0	3.0
LGMTPM04 Seg01T09	371	388	10	431	452	10	371.0	389.0	433.0	450.0	1.0	2.8	3.9	98.9	96.2	2.6	2.7
LGMTPM04 Seg01T10	373	389	9	433	450	9	375.0	389.3	430.3	451.0	2.0	2.9	3.2	92.8	95.0	2.2	2.4
LGMTPM04 Seg01T11	378	389	11	433	451	9	376.3	389.3	430.3	452.3	1.8	3.1	3.6	93.0	90.0	3.0	3.2
LGMTPM04 Seg01T12	375	389	9	432	455	9	377.8	389.0	430.3	451.0	2.8	4.4	4.9	91.2	96.1	4.9	5.3
LGMTPM04 Seg01T13	366	389	8	443	460	9	370.3	391.3	440.8	458.3	4.9	2.9	4.6	107.1	103.2	3.9	3.6
LGMTPM04 Seg01T14	370	390	10	439	454	10	372.5	392.3	438.5	451.0	3.4	3.0	4.8	98.3	96.1	2.2	2.2
LGMTPM04 Seg01T15	373	386	9	438	453	9	374.3	387.5	436.0	451.0	2.0	2.8	3.2	98.6	101.0	2.4	2.5
LGMTPM04 Seg01T16	372	391	9	430	452	11	375.3	389.5	430.3	451.0	3.7	1.0	3.3	92.5	93.9	1.4	1.5
LGMTPM04 Seg01T17	373	394	9	436	452	9	375.0	392.3	435.0	451.0	2.7	1.4	3.2	94.0	95.2	1.3	1.4
LGMTPM04 Seg01T18	378	389	9	435	450	9	375.3	386.0	430.3	451.0	4.1	4.9	3.5	95.2	92.0	3.1	3.3
LGMTPM04 Seg01T19	379	389	9	437	455	9	377.5	385.8	435.3	451.0	3.6	4.4	3.7	95.1	98.1	3.0	3.1
LGMTPM04 Seg01T20	378	389	9	438	453	9	377.9	388.0	436.3	451.0	1.0	2.7	3.9	95.9	96.1	0.2	0.2
LGMTPM04 Seg01T21	377	389	9	434	452	9	374.3	389.0	431.0	451.0	2.7	3.2	4.1	94.0	92.2	1.8	1.9
LGMTPM04 Seg01T22	370	387	9	439	448	9	369.3	386.0	440.3	451.0	1.3	3.3	5.1	106.3	102.0	4.3	4.0
LGMTPM04 Seg01T23	376	389	9	433	449	9	375.1	388.3	430.3	451.0	1.2	3.4	3.2	93.3	92.6	0.7	0.8
LGMTPM04 Seg01T24	372	389	9	433	449	9	375.3	389.3	430.3	451.0	3.3	3.4	4.0	92.6	92.7	0.1	0.1
LGMTPM03 Seg01T01	210	371	8	243	417	10	212.5	369.7	243.0	419.5	2.8	2.5	4.2	72.4	73.3	0.8	1.2
LGMTPM03 Seg01T02	215	372	9	245	419	11	213.3	370.0	246.5	421.5	2.6	2.9	3.9	71.3	73.6	2.3	3.2
LGMTPM03 Seg01T03	216	375	9	249	420	10	214.5	371.3	248.0	422.8	4.0	2.9	3.6	71.3	70.0	1.3	1.9
